# Reconstruction of Chronic Proximal Hamstring Tear: A Novel Surgical Technique with Semitendinosus Tendon Allograft Assisted with Autologous Plasma Rich in Growth Factors (PRGF)

**DOI:** 10.3390/jcm11185443

**Published:** 2022-09-16

**Authors:** Antonio Ríos Luna, Homid Fahandezh-Saddi Díaz, Manuel Villanueva Martínez, Ángel Bueno Horcajadas, Roberto Prado, Eduardo Anitua, Sabino Padilla

**Affiliations:** 1 Department of Traumatology and Orthopedic Surgery, Clínica Orthoindal, 04004 Almería, Spain; 2 Department of Orthopedic Surgery, Hospital Universitario Fundación Alcorcón, 28922 Alcorcón, Spain; 3 Department of Traumatology and Orthopedic Surgery, Avanfi Institute, 28015 Madrid, Spain; 4 Department of Radiology, Hospital Universitario Fundación Alcorcón, 28922 Alcorcón, Spain; 5 BTI—Biotechnology Institute I MAS D, 01007 Vitoria, Spain

**Keywords:** proximal hamstring avulsion, allograft reconstruction, chronic, PRGF, platelet-rich plasma, PRP

## Abstract

The reconstruction of a chronic proximal hamstring tear is a challenging pathology that posits difficulties to surgeons due to the distal retraction of the hamstring tendon stumps and the entrapment of the sciatic nerve within the scar formed around the torn hamstring tendon. We describe a novel surgical technique using a semitendinosus tendon allograft sutured in a “V inversion” manner, thereby avoiding an excess of tension and length of the new reconstructed hamstring tendons. In addition, and in order to speed up the healing process and avoid new sciatic entrapment, we assisted the surgery with liquid plasma rich in growth factors (PRGF) injected intraosseously, intratendinously and within the suture areas, as well as wrapping the sciatic nerve with a PRGF membrane. In conclusion, this novel approach offers mechanical and biological advantages to tackle the large retraction of hamstring stumps and the entrapment of the sciatic nerve within the scar.

## 1. Introduction

A chronic proximal hamstring tear is a debilitating muscle injury associated with persistent pain, cramping, functional limitation, and weakness in the ischial (buttock) region, with more than 4–6 weeks of evolution [1]. The delayed diagnosis and the failed conservative treatments often result in a hamstring syndrome caused by the entrapment of the sciatic nerve within the scar formed around the torn hamstring tendon, causing an unrelenting pain that is exacerbated by prolonged sitting [2,3]. Moreover, the distal retraction of the hamstring tendon stumps of several centimeters makes the reinsertion of the entire conjoined tendon (JT) (made up of the long head of biceps femoris (LBF) and semitendinosus tendons (STT)) and the tendon of semimembranous muscle (SMT) extremely difficult [1,2].

In this manuscript, we describe a novel technique using a semitendinosus tendon allograft sutured in a “V inversion” manner, thereby avoiding an excess of tension and length of the new reconstructed hamstring tendons, which could lead to rupture at the suture areas. Moreover, and in order to enhance the repair process and avoid new sciatic entrapment, we assist the surgery with liquid plasma rich in growth factors (PRGF-Endoret) injected intraosseously, intratendinously, and within the suture areas, as well as wrapping the sciatic nerve with a PRGF membrane [4,5].

## 2. Case Report and Description of the Technique

### 2.1. Patient Presentation and Examination

**Figure 1:**
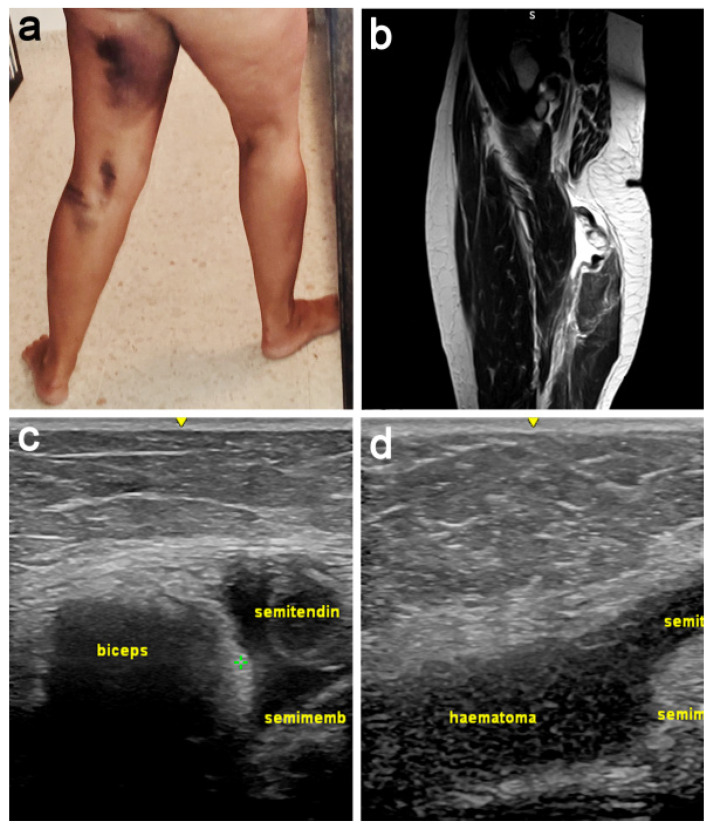
Preoperative description of the case. (**a**) Image of the haematoma two days after the accident, accompanied by lancinating pain with paresthesia similar to radicular pain. (**b**) MRI confirming the injury; the conjoint tendon was retracted 7 cm and the semimembranosus tendon 10 cm, with a significant accumulation of free fluid. (**c**,**d**) Ultrasound also showed complete disinsertion of both tendons, as well as their retraction.

A 45-year-old female with no medical history relevant to this injury underwent a motorcycle fall at low speed with the left lower extremity in hyperextension. She heard a snap, together with an acute intense cramping pain from the buttock region to the knee. Once at the hospital, the X-ray study did not show a bone fracture. She began a physiotherapy treatment, but, after a few days, the patient noticed a significant hematoma associated with a persistent pain (Figure 1a).

In this second visit to the hospital, the performed US and MRI studies confirmed a complete detachment of conjoined tendon of biceps femoris and semitendinosus (JT), and the semimembranous tendon (SMT). After following a conservative treatment and 6 months after the accident, the patient came for the first time to our consultation reporting a significant functional limitation. Physical examination showed a limitation in knee extension while walking, a gap distal to the left gluteal fold, and the impossibility of knee flexion against resistance with a positive Puranen–Orava test. New MRI (Figure 1b) and US (Figure 1c,d) studies showed that the JT was retracted 7 cm and the SMT 10 cm. This injury could be classified as type 5-B following the classification developed by Wood et al. [6], namely, a complete tendon avulsion from bone with a retraction of the tendon ends associated with sciatic nerve involvement.

After discussing treatment options and possible outcomes, the patient underwent a surgical intervention with a novel technique using a semitendinosus tendon allograft in a “V inversion” manner assisted with liquid PRGF injected intraosseous, intratendinously and within the suture areas, and a PRGF membrane that wrapped the sciatic nerve.

### 2.2. Patient Positioning

**Figure 2:**
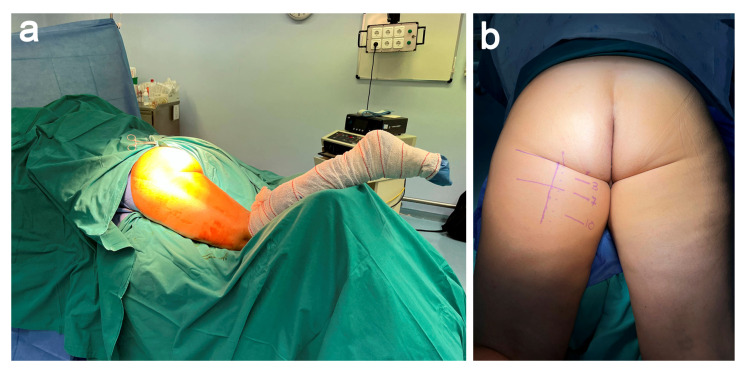
(**a**) The patient is placed in prone position with the hip and knee flexed. (**b**) Previously, the relevant anatomical structures are located.

After the induction of general anesthesia, the surgery was performed with the patient in the prone position, with the hip and knee at 20 and 30 degrees of flexion, respectively (Figure 2a). Previously, and in order to center the incision, we identified the stumps of the conjoined tendon (Biceps and semitendinosus) and SMT stumps with the aid of US and MRI (Figure 2b). In our patient, the distance between the ischial tuberosity and the gluteus fold was 3 cm, whereas the joint tendon and SMT were 7 and 10 cm away from the ischial tuberosity, respectively. Once the sterile prepping and draping were completed, the entire extremity was draped so that we could manipulate the hip and knee joints during the surgery to assess and adjust the graft tensioning.

### 2.3. Dissection, Neurolysis, and Tenolysis

**Figure 3:**
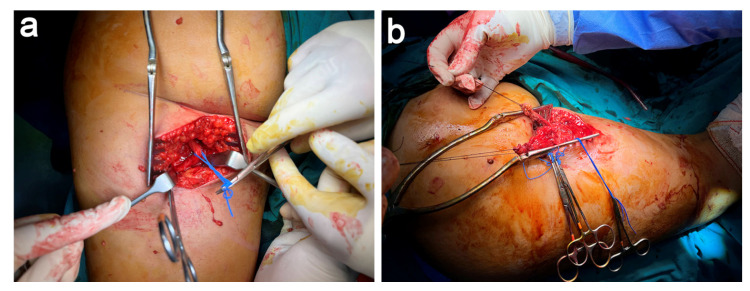
Dissection, neurolysis, and tenolysis (**a**) After exoneurolysis, the sciatic nerve is identified and placed laterally in the surgical field. (**b**) Following tenolysis, Vycril sutures are placed for traction on both tendons.

A longitudinal/vertical incision was performed guided by the landmarks obtained with the aid of US and MRI. After dissecting the subcutaneous tissues, we identified the ischial tuberosity, torn hamstring tendons, and the entrapped sciatic nerve (Figure 3a). We carried out a careful distal-to-proximal exoneurolysis of the sciatic nerve and placed vessel-loops to visualize and avoid iatrogenic injury to the nerve during the surgery. Once identifying the stumps of both tendons that were retracted and entrapped by fibrotic tissue, we performed a careful tenolysis to free up both tendon stumps and freshen both stumps. In addition, we put Vicryl (Ethicon, Somerville, NJ, USA) sutures in each tendon stump, which served as control of the torn hamstring (Figure 3b).

### 2.4. STT Allograft Preparation

**Figure 4:**
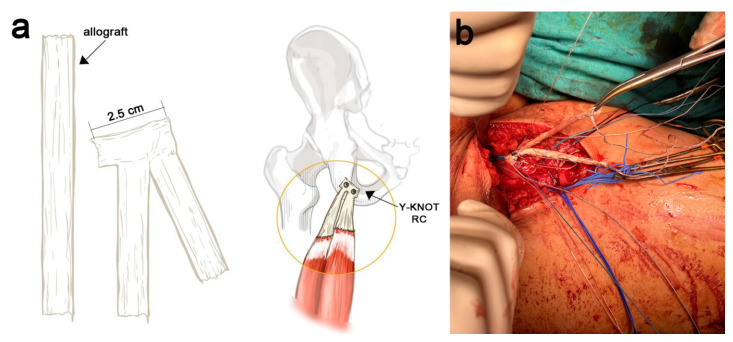
Semitendinosus tendon allograft preparation. (**a**) Description of allograft preparation and placement. (**b**) Intraoperative image showing the two branches of the allograft (inverted V-shaped).

Due to the impossibility of reinserting the hamstring tendon stumps into the ischial tuberosity, a free graft reconstruction with a 17 cm long STT allograft augmentation was carried out, allowing us to bridge the long gap (Figure 4a). The entire STT allograft was reinforced with a Hi-Fi Ribbon suture (ConMed, Largo, FL, USA), thereby endowing the reconstructed tendon augmentation with additional strength. The STT allograft was bent on itself, generating a double-thickness tendon allograft whose proximal section of 3 cm was reinforced with 2/0 Ethibond sutures (Ethicon, Somerville, NJ, USA), from which, were the two branches of the allograft stem toward the distal hamstring stumps in a “V inversion” shape (Figure 4b).

### 2.5. STT Allograft Placement

**Figure 5:**
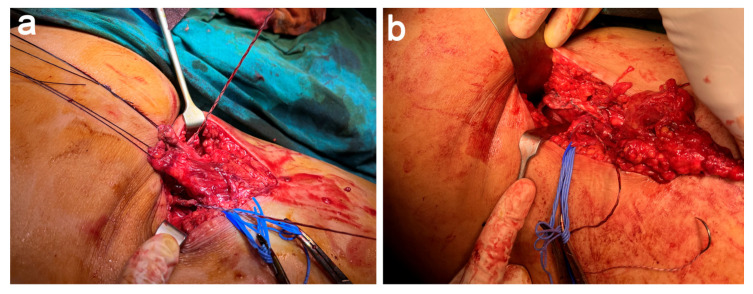
Semitendinosus tendon allograft placement. (**a**) Support with Ribbon resistance band. (**b**) Allograft to tendon suture.

After identification, dissection, and protection of the sciatic nerve, and following the correct anatomically location, we prepared the bony surface of the ischial tuberosity by curettage and rasp. Then, two self-punching Y-Knot RC (ConMed, Largo, FL, USA) suture anchors (two Hi-Fi, one blue and one white) were placed in the ischial tuberosity (Figure 5a). The first Y-Knot RC anchor was placed in a lateral and anterior position (corresponding to the SMT that is lateral and 3.1 cm proximal-to-distal and 1.1 cm medial-to-lateral). The second Y-Knot RC anchor was placed in a more posterior and medial location (the JT is medial 2.7 cm proximal-to-distal and 1.8 cm medial-to-lateral).

Krakrow type stitches were passed with the blue strand of a Y-Knot RC in the external arm of the “V” of the double reinforcement of the allograft. We performed the same but using the white strand at the base of the medial arm of the “V”. As it is a sliding thread, by pulling on another thread of the same color that remains free, the graft slides and is placed in the anatomical position at the level of the ischial tuberosity. Once this double suture is secured, we made a medial and lateral reinforcement suture with the blue and white threads left over in each Y-Knot RC to provide significant solidity in the insertional area.

We then took the two tendon ends: the joint tendon on one side and the semimembranosus tendon on the other side. Next, we passed one of the distal ends of the allograft through the semimembranosus tendon (deeper) with a Pulvertaft-type suture. We checked the tension after the two passes, and we carefully mobilized the knee in a range of 60 degrees, which allowed us to assess the right tension of our suture. After verifying that it was satisfactory, we made two more passes to provide a strong suture. We repeated the procedure with the joint tendon and performed four tendon passes with the Pulvertaft-type technique (Figure 5b).

### 2.6. PRGF Preparation and Application

Platelet-rich plasma (PRP) was prepared according to the PRGF-Endoret method. PRGF is a leukocyte- and erythrocyte-free PRP with moderate platelet-enrichment [7]. Briefly, 72 mL of peripheral venous blood was withdrawn into 9 mL tubes containing 3.8% (wt/vol) sodium citrate (Endoret Traumatology kit, BTI Biotechnology Institute, Vitoria, Spain) before starting the patient’s anesthesia. Then, the blood was centrifuged for 8 min at 580 g at room temperature in a System V centrifuge (BTI Biotechnology Institute, Vitoria, Spain). After centrifugation, three layers were obtained (plasma, buffy coat and packed red blood cells). The upper layer of plasma (F1 fraction) was collected in order to prepare the PRGF membrane. The 2 mL plasma fraction located just above the buffy coat (F2 fraction) was collected, avoiding the leukocyte layer, and was used to perform infiltrations.

**Figure 6:**
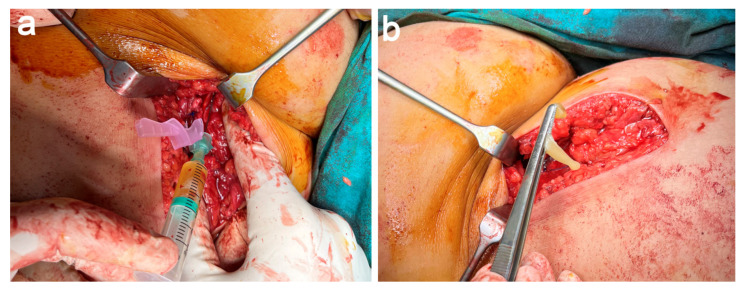
Application of PRGF. (**a**) Intratendinous infiltration of PRGF into the allograft suture-repair areas. (**b**) The sciatic nerve is wrapped with a PRGF membrane.

We performed intraosseous (4 mL into the ischial tuberosity once freshened) and intratendinous infiltrations (8 mL) of liquid PRGF into the suture-repair areas (Figure 6a) The F2 fraction was activated in a time-controlled way by the addition of PRGF activator (10% CaCl_2_) just before to these infiltrations performed with a 21 G − 0.8 × 40 mm needle. Finally, the sciatic nerve was wrapped with a PRGF membrane (Figure 6b) elaborated with 8 mL of liquid F1, which was activated with 160 μL of PRGF activator and maintained for at least 15 min at room temperature until the formation of a clot.

Furthermore, seven and fourteen days after the surgery, and assisted by US, we performed two infiltrations of PRGF in the sutured areas of the graft. In both cases, 8 mL of freshly activated F2 PRGF was infiltrated [4,5].

### 2.7. Postoperative Rehabilitation

**Figure 7:**
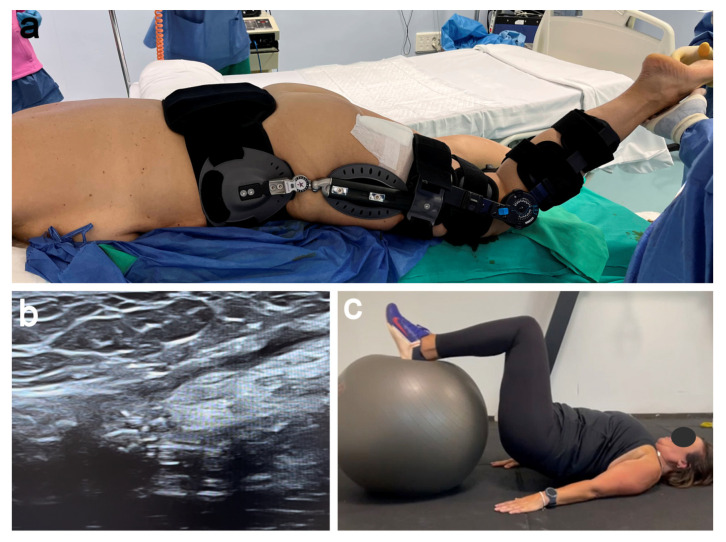
Postoperative rehabilitation. (**a**) Immobilizing splints keeping the knee and hip flexed. (**b**) US (week 12 postoperatively) showed images compatible with the integration of the allograft. (**c**) Rehabilitation process starting at 12 weeks.

The patient was kept in a hip and knee brace locked at 70 degrees and 30 degrees of flexion, respectively, for 8 weeks, not allowing the weight-bearing walk and using two crutches (Figure 7a). After 8 weeks, we removed the hip and knee brace and the patient underwent a rehabilitation program with a passive progressive range of motion of the hip and knee, allowing partial weight-bearing using two crutches. The US performed at week 12 showed images compatible with the integration of the allograft into the tendon (Figure 7b), which was the start point of an active rehabilitation program (Figure 7c). In a gradual manner, and always supervised by the physiotherapist and surgeon advice, our patient initiated quadriceps and hamstring isometric, eccentric and proprioceptive exercises, as well as active resistance strength exercises. After 12 months, the patient resumed her active lifestyle without any limitation.

## 3. Discussion

We describe the reconstruction of a chronic proximal hamstring tear using a semitendinosus tendon allograft sutured in a “V inversion” manner and assisted with PRGF as a novel surgical technique to treat a chronic proximal hamstring tear. Our patient resumed her active lifestyle without any limitation 12 months after the surgery. This new technique offers mechanical and biological advantages to tackle the large retraction of hamstring stumps and the entrapment of the sciatic nerve within the scar. In fact, 9 months after the surgery, the patient resumed her previous lifestyle, including recreational sport.

There have been reported numerous different procedures to overcome and bridge tear gaps superior to 5 cm between the tendon stumps and the ischial tuberosity in chronic proximal hamstring tears by using ipsilateral distal hamstring autografts [8] or Achilles tendon allografts [9], both with good post-operative clinical outcomes and patient satisfaction with 24 and 48-month (long term) follow-ups. Despite some inherent potential drawback of allografts, including infection and disease transmission, issues with the osseointegration and the cost and shortage of allografts, the surgical repair of retracted stumps superior to 5 cm is recommended and often necessary [2,9]. We chose to use a semitendinosus tendon allograft of 17 cm due to the dimensions of the stump retractions of 7 and 10 cm. In doing so, it endowed the reconstructed tendon augmentation with a controlled suture tension and the correct anatomically location at the proximal insertion, thereby recreating the native insertion at the ischial tuberosity [2]. Significantly, the Y-Knot RC anchor associated with robust sutures allow loads superior to 200 N, and having this type of fixation emerged as the gold standard treatment [10]. Moreover, the longitudinal/vertical incision guided by the landmarks obtained with the aid of US and MRI gave us enough room to perform the surgery accurately, assessing the tension of the suture anchors and the sutured stumps, as well as the use of PRGF in different surgical steps. The PRGF supplied the suture anchor and sutured stumps with trophic molecules that have been reported to promote the osseointegration of the graft, a better remodeling and the secretion of extracellular matrix, while avoiding fibrosis at the suture stumps as well as around the sciatic nerve, all effects leading to enhance the repair process [4,11,12,13]. At this point, and following Sanchez et al. [14], one improvement to add would be to soak in and infiltrate the allograft into the PRGF supernatant. However, this novel technique is not exempt from some pitfalls, mainly stemming from the long vertical incision and the period of 8 weeks wearing the hip and knee brace, the latter being cumbersome and hard to tolerate for patients. We consider that it is not recommended to shorten this time, as the immobilization time depends on the type of tendon sutured, the type of suture and whether or not allografting is required. In our case, these are very powerful tendons that require and demand a lot of strength, and the time described should be respected to avoid the risk of dehiscence or suture failure. A long longitudinal incision is recommended in chronic proximal hamstring tears when the tear distal gap of the hamstring tendon stumps assessed by US and MRI is several centimeters [2].

## 4. Conclusions

The PRGF-assisted reconstruction of chronic proximal hamstring tears with a semitendinosus tendon allograft provides mechanical and biological advantages to tackle the large retraction of hamstring stumps and the entrapment of the sciatic nerve within the scar.

## Data Availability

Not applicable.

## Abbreviations

JT	Conjoined tendon
LBF	Long head of biceps femoris
PRGF	Plasma Rich in Growth Factors
PRP	Platelet-rich plasma
SMT	Tendon of semimembranous muscle
STT	Semitendinosus tendon
